# One-Dimensional NMR Imaging of High-Temperature First-Drying in Monolithics

**DOI:** 10.1007/s00723-018-1018-x

**Published:** 2018-05-18

**Authors:** A. J. Barakat, L. Pel, O. C. G. Adan

**Affiliations:** 1 Materials Innovation Institute M2i, Elektronicaweg 25, 2628 XG Delft, The Netherlands; 2 Eindhoven University of Technology, De Rondom 70, 5612 AP Eindhoven, The Netherlands; 3 TNO, High Tech Campus 25, 5656 AE Eindhoven, The Netherlands

## Abstract

In this study a specialized high-temperature nuclear magnetic resonance (NMR) setup is presented for measuring free moisture in monolithic refractory castables during one-sided heating (100–300 °C). This setup makes use of a high thermal-stability Birdcage-coil for measuring the quantitative moisture content at high-temperatures, while also utilizing a mini-coil for calibrating transverse relaxation changes, as a function of temperature and hydration state, taking place in the sample throughout a drying experiment. We employ a high-temperature correction scheme that calibrates the effects of rising temperatures on the NMR signal. With this configuration, we can non-destructively measure moisture and temperature profiles continuously and achieve a spatial resolution of 2–3 mm for samples as long as 74 mm. After applying the NMR correction, we can extract information about the physical and chemical components of water as they are released from the porous matrix during first heat up. As a model material, we demonstrate the capability of our setup with a conventional castable after it has been cast and cured for 48 h.

## Introduction

In steel and iron processing plants, the hot ladles where these metals are transported must be lined with refractories. These materials are capable of withstanding physical, thermal and chemical stresses and strains over a wide range of temperatures [1, 2]. In contrast to brick (shaped refractories), the installation and maintenance of unshaped monolithic refractories (i.e. castables) require less labour costs and fewer repairs since they have a longer service lifetime. It is for this reason that monolithics (of which castables constitute half of production output) make up more than twice the production level of bricks in Japan, for instance [3].

However, prior to installation, the refractory elements must be slowly pre-dried at less than 1 °C/min for at least 24 h to temperatures higher than 500 °C, in order to remove the free and chemically bound water components from the porous matrix.

The conservative heating rates are necessary due to generated steam pressures within the pores that soar rapidly beyond the mechanical strength of the material (2–6 MPa), sometimes causing physical destruction via explosive spalling. While these drying temperatures are quite high for laboratory measurements, it is known that the heightened risk of spalling is actually found in both the dangerous so-called ebullition stage (100–300 °C) and hydrate decomposition stage (250–300 °C). In fact, damage can actually occur at these relatively low temperatures. Therefore, a quantitative understanding of the transport dynamics in the regime of first-drying (100–300 °C) is paramount to optimizing the durability and lifetime of these materials for industrial applications. With current global steel production output exceeding 1000 million tons per year [4], there is great urgency for improving the design and performance of castables. Therefore, increasing the heating rate, altering the drying behaviour through control of material inputs and minimizing service failure is of great economic concern for industrial producers.

In order to assess the potential of spalling, indirect and destructive methods are often employed to characterize the phase morphology (XRD), dehydration and mass loss of water (TGA/DSC). Unfortunately, all of these methods suffer from limitations on information that can be obtained due to inadequate sample size (preventing development of a thermal gradient) or sample destruction (i.e. by crushing to grains/powder). Evermore, measuring the mass loss over time only provides information about the total moisture content, without providing any direct observation of the spatially resolved moisture distribution. More importantly, crucial information for calculating transport properties, such as the speed and position of the drying front, as well as the corresponding vapour pressure, is completely lost on such conventional experimental methods. While the use of neutron and X-ray radiography can also provide information about the moisture distribution, these techniques are limited by the maximum sample size, boundary artifacts and inability to distinguish between the different components of water [5].

Modelling the transport dynamics is another alternative for quantifying the moisture distribution, but this relies on either second-guessing the behaviour of drying (e.g. advection vs. diffusion) or introducing simplifying assumptions (e.g. single phase vs. multiphase phenomena) into the underlying theory [6, 7].

In light of these constraints, a new experimental approach is in order that incorporates the need for direct, non-destructive and continuous measurement of the moisture and temperature distribution throughout a first-drying experiment. The goal of this paper is to demonstrate that the quantitative moisture content can be directly measured by Nuclear Magnetic Resonance (NMR) and is related to the drying behaviour for high-temperatures without compromising the sample.

Previous work on quantifying moisture loss with NMR focused on materials (e.g. concrete) that have already aged for 1 full year [8]. At this point curing conditions become less determinate of pore structure, which is almost fixed in time for low temperatures. In practice, however, castables must be freshly cured, pre-dried and then immediately installed for service. Therefore, unlike concrete, the castable pore structure is continuously developing and changing, starting at room temperature until 100°C [9]. As a result, we had to perform a correction of the signal for both the temperature and the pore-size development based on the measured *T*_2_. Furthermore, castables are suitable materials to study under NMR as their composition is abundant in aluminum oxide, thereby containing very little paramagnetic impurities (e.g. iron oxide).

In the next section, we will present the outline for an experimental NMR setup which is capable of performing quantitative moisture content measurements at high temperatures on a sample material. To this end, we use conventional castables (CC) that can be formulated in the lab and scanned inside the NMR insert for continuous periods of time. The temperature calibration of the NMR signal will also be presented and discussed at length.

## Experimental Setup

**Fig. 1:**
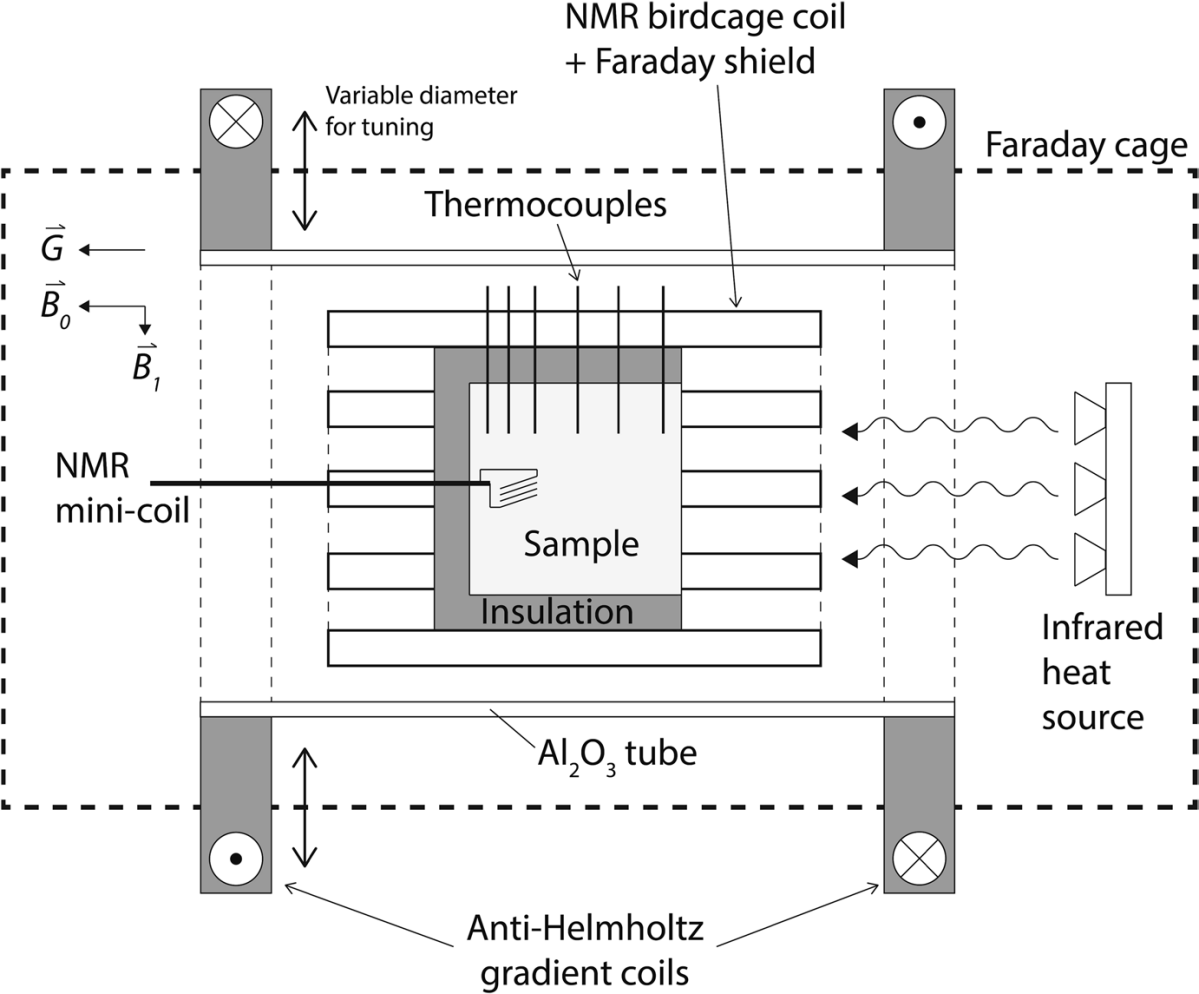
Schematic view of the high-temperature NMR setup. The birdcage coil is enclosed by a pair of anti-Helmholtz gradient coils with a calibrated strength of 80 mT/m and a Faraday cage for tuning and matching. The heat source is provided by an array of seven halogen lamps

A home-built NMR setup was used for performing one-sided, non-isothermal first-drying experiments on conventional castables. In order to perform these measurements we use the main magnetic field of a whole-body MRI scanner. A schematic view of the setup is presented in Fig. 1. The experiments are conducted inside a Philips whole-body 1.5 T magnet (INTERA). The samples are cast into 8 cm-diameter cylindrical Teflon moulds (preventing moisture loss, with exception to the front face which remains open to heating) and additionally isolated with 5 cm thick and 8 cm long rock wool in the same manner to prevent heat loss during an experiment. In this way, we can simulate one-dimensional heating (as in a real world setting) allowing for the development of a moderate temperature gradient in one direction.

**Fig. 2:**
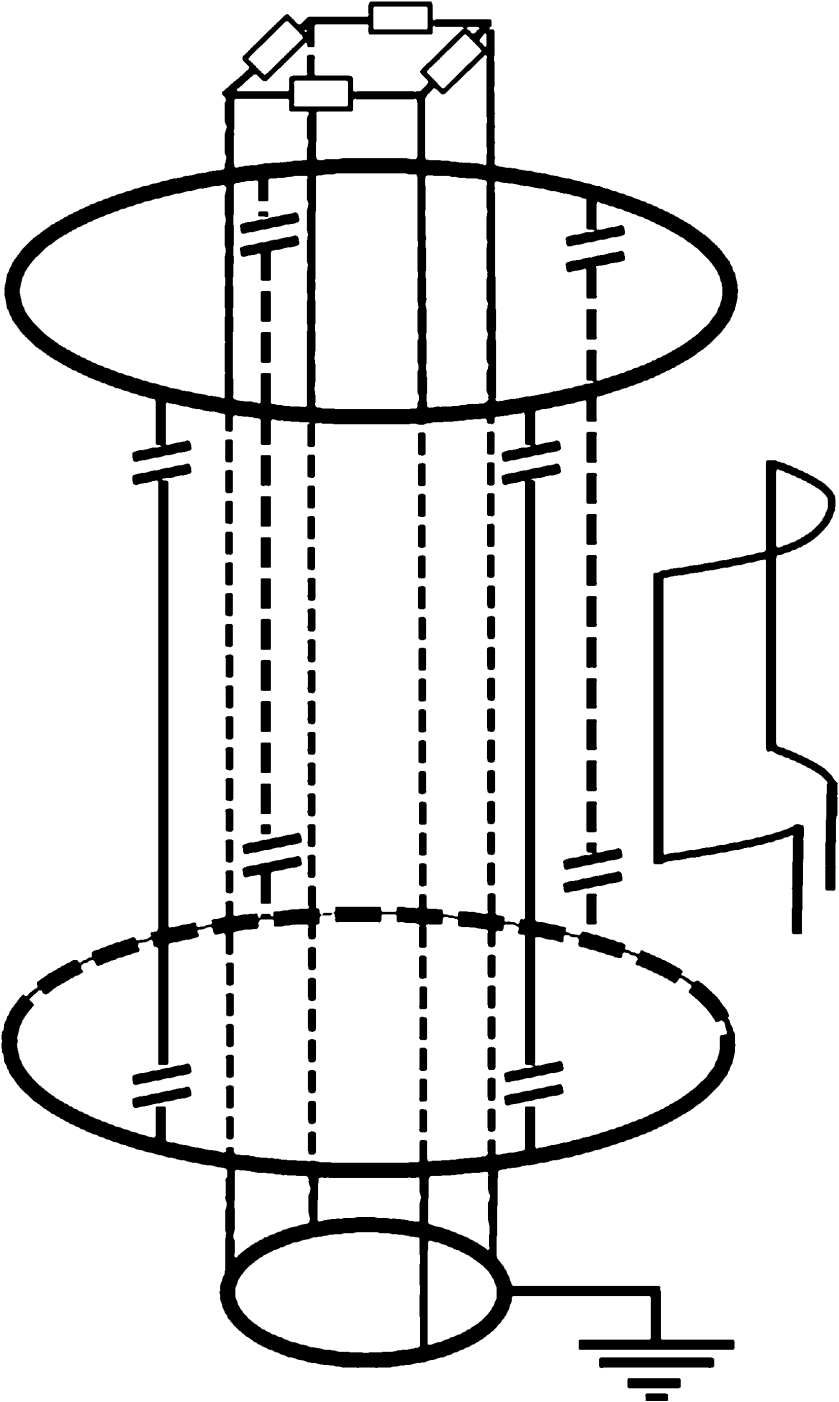
A schematic diagram of the home-built low-pass birdcage. The conducting segments of the birdcage are excited by an inductive coupling loop. A shielding coil between the sample and RF Birdcage coil is inserted to prevent detuning, in order to minimize the effect of dielectric variations. The other side of the legs are interconnected through a small resistance (1 kΩ), effectively acting as a load to decrease the Q (i.e. of the LC-circuit to about 40)

As we aim to perform quantitative measurements over the full temperature range up to 300 °C, special attention was given to the design of the birdcage coil. Here we make use of a home-built low-pass 64 MHz RF homogeneous birdcage resonator for conducting pulsed measurements. The birdcage is built around an Al_2_O_3_ tube capable of withstanding high temperatures, without introducing a background signal to the NMR. A schematic diagram of the birdcage used in these experiments is presented in Fig. 2. In order to make sure the birdcage does not detune excessively during the measurements, as both the temperature and moisture content are changing, we added a shielding coil between the sample and RF birdcage coil. This coil consists of electrically grounded and interconnected conducting segments running parallel to the legs of the birdcage resonator [10] (also see Figs. 1 and 2). On one side these are connected to the ground to prevent as much as possible any dielectric variations, due to the changing moisture content. In addition, the other side of the legs are interconnected through a small resistance (1 kΩ), which means that this coil will also act as a load, decreasing the *Q* of the LC-circuit to about 40. In combination with the low filling factor, this makes the system capable of performing quantitative measurements of the mass loss.

The birdcage itself is enclosed by a second shield composed of a copper mesh. The function of this shield is two-fold: protecting the MRI magnet in case of an explosion from the sample and tuning the resonance frequency of the birdcage by changing the diameter of the shield. The resonator is related to the spectrometer through an inductive coupling loop opposing the conducting segments of the probe. With this current setup we are able to generate a Hahn spin-echo with a pulse time of 50 μs using a 4-kW homebuilt power amplifier. The shortest echo time we can achieve is 300 μs. For such a sequence, a block-shaped pulse is employed for exciting the sample protons.

In order to perform profile measurements, two anti-Helmholtz magnetic field gradient coils are used with a maximum strength of 80 mT/m. With this maximum gradient, we can achieve a one-dimensional resolution on the order of 2–3 mm for the castables considered. With 25 slices, a single-slice excitation imaging technique is used in conjunction with the Hahn pulse sequence described above for measuring one-dimensional profiles. As the bandwidth of the insert is 250–300 kHz, the moisture profile is measured by changing the resonance frequency, i.e. during our experiment we do not switch the gradient. Furthermore, because the castable’s *T*_1_ is on the order of a few tens of milliseconds, using a Hahn spin echo excitation scheme allows for measuring a complete profile over 100 mm within 5–10 min.

In order to get a better idea of how the relaxation time of our sample is changing with time, a solenoidal mini-coil of 10 mm diameter was added to the back of each sample (which is accomplished by casting). The details of this installation procedure are explained more precisely in Sect. 4.1. Due to the significantly smaller scanning volume (of the mini-coil) and greater filling factor (10-fold greater than the birdcage coil), a better signal-to-noise ratio enabling faster measuring time was achieved. Furthermore, with such a tiny probe in place, a short echo time of 110 μs is achieved that allows us to measure the shorter *T*_2_ components as a function of temperature. The gathered *T*_2_ data are used for calibrating the temperature effect at every slice of the sample. Since the samples are homogeneous, performing a CPMG measurement at every slice is not necessary. A practical advantage of this approach is that it allows positioning the coil perpendicular to the main field, ensuring a 90° rotation of the spins regardless of the orientation of the sample. It was found that this mini-coil had almost no effect on the performance of the main birdcage.

When casting materials, the samples are punctured with holes accommodating thermocouple placement for temperature profile measurements, which are also necessary for correcting the temperature effects on the NMR signal. Using a template scale, 6 thermocouples are non-linearly fixed into the sample holder, starting from front to back: 7, 27, 44, 56, 64 and 71 mm. The RF mini-coil is located at the same position as the last thermocouple (71 mm). The heat source for the experiments is provided by an array of seven gold-coated halogen lamps facing the front of the sample (with a corresponding heat flux on the order of 3.5 kW/m^2^ and a dissipated power of 100 W per lamp). The maximum heating rate was found to be on the order of 1–2 °C/min in the present setup.

## NMR Signal Temperature Dependence

### Temperature Influence

In a high-temperature NMR experiment, the temperature will have a profound effect on both the relaxation and the magnetization. In which case, the standard expression for the magnetization must be rewritten as follows:

1 $$M\left( {t_{\text{E}} ,t_{\text{R}} ,T} \right) = M_{0} (T)\exp \left( { - \frac{{t_{\text{E}} }}{{T_{2} \left( T \right)}}} \right)\left[ {1 - \exp \left( { - \frac{{t_{\text{R}} }}{{T_{1} \left( T \right)}}} \right)} \right],$$

where *M* is the time-dependent magnetization, *t*_*E*_ the Hahn spin-echo time, *t*_*R*_ the repetition time and *T*_1_ and *T*_2_ correspond to longitudinal and transverse relaxation, respectively. The equilibrium magnetization $M_{0}$ will scale inversely proportional to the absolute temperature *T* of the field as given by the well-known Curie law, i.e. [11]:

2 $$M_{0} (T) = \frac{{N\mu \hbar \gamma B_{0} }}{2kT} ,$$

where *N* is the number of nuclei, $\mu$ the magnetic moment, $\hbar$ the Planck constant, $\gamma$ the gyromagnetic ratio, *B*_*0*_ the magnetic field and *k* the Boltzmann constant.

**Fig. 3:**
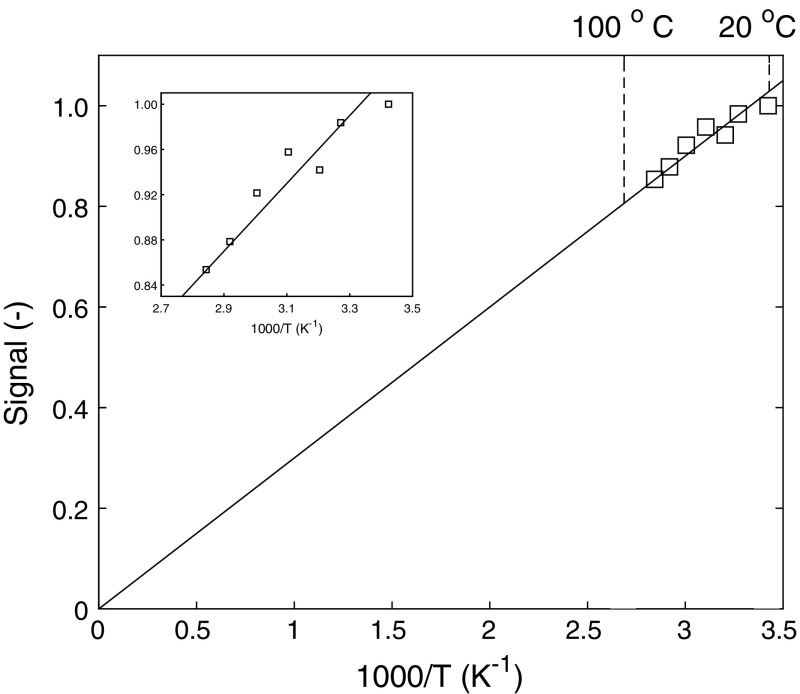
The measured NMR signal amplitude of water as a function of reciprocal temperature. The straight line demonstrates the Curie law from 20 to 100 °C

In Fig. 3 we have provided the results for a measurement of the signal of water as a function of temperature by heating a small container of water up to the boiling point, inside our experimental setup. As can be seen, the relationship between the signal and reciprocal temperature is linear, in accordance with the Curie law.

By partially differentiating the logarithm of the magnetization with respect to temperature, we can decompose the separate effects of various mechanisms on the temperature-dependence of the measured signal [12]:

3 $$\frac{{\partial ln M\left( {t_{\text{E}} ,t_{\text{R}} ,T} \right)}}{\partial T} = \frac{{\partial ln M_{0} }}{\partial T}{ + } \, \frac{{t_{\text{E}} }}{{T_{2}^{2} \left( T \right)}}\frac{{\partial T_{2} \left( T \right)}}{\partial T} - \frac{{t_{\text{R}} }}{{T_{1}^{2} (T)}}\frac{{\exp \left( { - \frac{{t_{\text{R}} }}{{T_{1} \left( T \right)}}} \right)}}{{1 - \exp \left( { - \frac{{t{\text{R}}}}{{T_{1} \left( T \right)}}} \right)}}\frac{{\partial T_{1} (T)}}{\partial T}.$$

Here we see the main contributions to the temperature dependence are due to magnetization and *T*_1_ and *T*_2_ relaxation. If we set *t*_R >>_ *T*_1_ in our experiments, we can then neglect the *T*_1_ dependence to get:

4 $$\frac{{\partial ln M\left( {t_{\text{E}} ,t_{\text{R}} ,T} \right)}}{\partial T} \approx \frac{{\partial ln M_{0} }}{\partial T}{ + } \, \frac{{t_{\text{E}} }}{{T_{2}^{2} \left( T \right)}}\frac{{\partial T_{2} \left( T \right)}}{\partial T}.$$

Hence we see that the temperature correction is a function of both the echo-time and the spin–spin relaxation time of our sample. Hence for a fixed *t*_E_ any change in *T*_2_ will result in a change of the NMR signal.

### Relaxation Correction

During an NMR experiment, the water molecules in a porous material will diffusively probe the pore space over a certain amount of time. As a result, relaxation will be dependent on the pore geometries and spin–spin relaxation can be described in the fast diffusion regime according to the well-known Brownstein-Tarr model (1979), i.e.:

5 $$\frac{1}{{T_{2} }} = \frac{1}{{T_{{2,{\text{Bulk}}}} }} + \rho_{2} \frac{S}{V},$$

where *ρ*_2_ is the surface relaxivity, *S/V* the surface-to-volume ratio and *T*_2,bulk_ the bulk relaxation for water. In most conventional castables the pores are in the range of 10-100 nm. In this length scale, *T*_2_ relaxation will consequently be dominated by surface relaxation. Accordingly, we can neglect the contribution of bulk relaxation. For comparison, gypsum has a pore-size distribution on the order of 1 μm and will, therefore, be dominated by slow-diffusion in the pores [13].

In our drying experiments, the relaxation will change due to the transforming pore-size distribution as the materials are hydrating. Furthermore, the temperature-dependence of the surface relaxivity will also cause the relaxation to change. The latter effect can be phenomenologically described by an Arrhenius-type equation [14, 15]:

6 $$\rho_{2} = \rho_{2,0} \exp \left( {\frac{\Delta E}{RT}} \right)$$

Here *ρ*_2,0_ is the surface relaxivity at a certain reference temperature, and Δ*E* is an effective surface interaction energy. The surface interaction energy is a combination of both the energies involved in surface relaxation and surface diffusion [14].

**Fig. 4:**
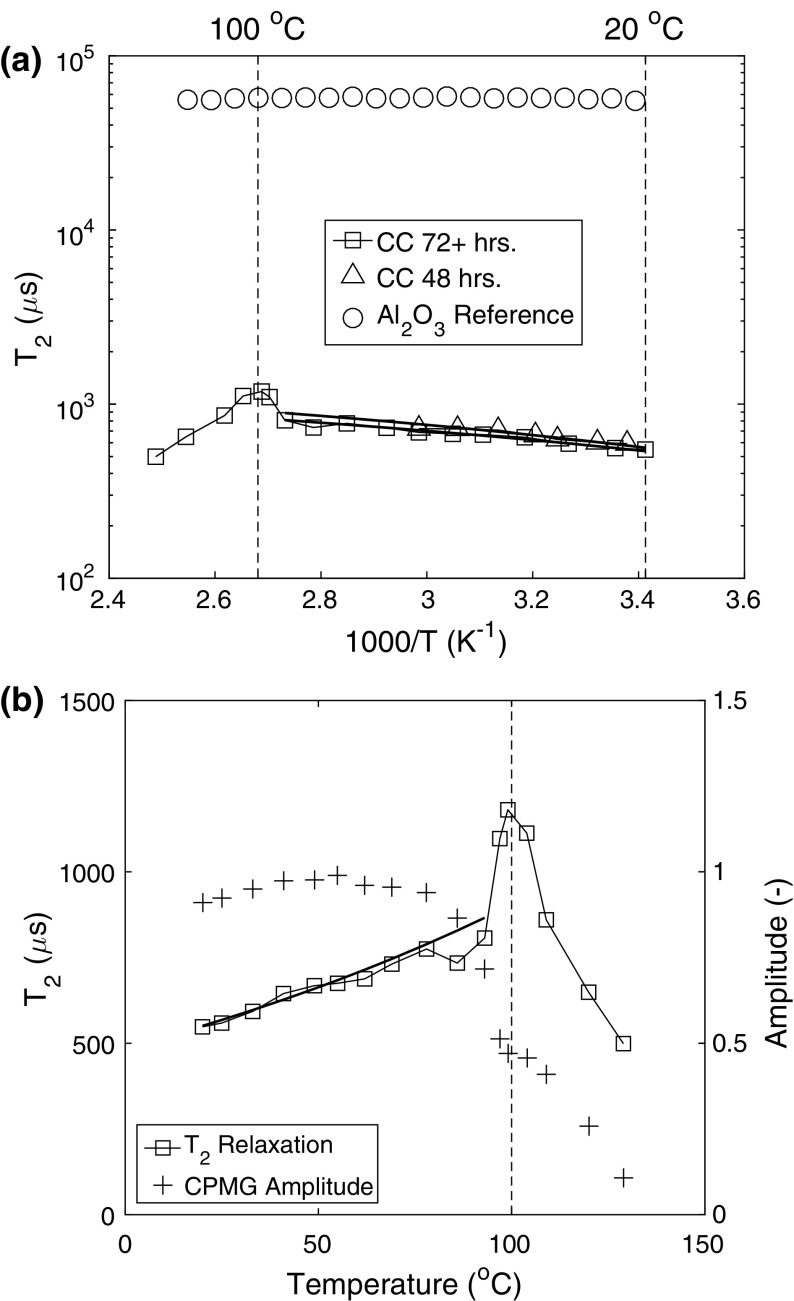
**a** The T_2_ as measured with the mini-coil plotted as a function of reciprocal temperature for conventional castables cured for 48 and 72 h, along with an aluminum oxide reference sample. The straight lines are a fit of the Arrhenius temperature-dependence. **b** The T_2_ measured for conventional castables cured for 72 h from (**a**) plotted against a normal temperature scale. The straight line is a fit to the Arrhenius temperature-dependence as given in **a**. The corresponding CPMG amplitude in relation to T_2_ is also plotted in the same figure

In Fig. 4 the measured relaxation times as a function of reciprocal temperature are demonstrated for two castables of different hydration time and an aluminum oxide reference material. As can be seen, the initial increase of the relaxation times can be described by an Arrhenius equation. From these measurements we find that in the case of castables *∆E *= 4-5 kJ/mol, which is of the same order as previously found for concrete *∆E* = 1-5 kJ/mol [15]. Here it has to be taken into account that for castables *T*_2_ will not only be a function of the temperature but also the hydration state, since the pore structure is not chemically inert with temperature.

The metastable hydrate phases are changing from room temperature until the boiling point of water, thereby redefining the structure of the solid network. However, around the boiling point, the molecules experience a phase change from liquid to vapour, followed by evacuation from the pores and a corresponding decrease in *T*_2_ as can be seen in Fig. 4A and B. It is also at this point that *T*_2_ spikes to a maximum value for a short time before the moisture evaporates. The moisture at this juncture is relaxing slower than it was before being heated. The exact cause for this spiking behaviour is not fully understood at the moment. One possible reason is that heated moisture is less bound to the confined geometry of the pores, which is likely since in this temperature range the porosity may also change and the viscosity is significantly lower than before (30 per cent of the initial value at room temperature). In Fig. 4B we have also plotted the corresponding CPMG amplitude as a function of temperature. As can also be seen at this point, around 100 °C, the CPMG amplitude temporarily levels off as *T*_2_* → T*_2,max_. Furthermore, between the temperatures 90 and 100 °C, *T*_2_ is clearly increasing to a maximum value while the corresponding CPMG amplitude is decreasing. This suggests that in the fast-diffusion regime, either the surface relaxivity or surface area of the pore must be simultaneously changing along with the volume fraction of moisture.

Furthermore, the spontaneous jump in $T_{2}$ cannot be attributed to just free water relaxing in the pores. To test this hypothesis, a chemically inert reference sample, i.e. aluminum oxide, with a uniform pore size distribution (on the order of 200 nm) was taken and capillary saturated for 24 h, before being heated under a heating program similar to the one used in the experiments considered here as shown in Fig. 4a. The results yielded a constant *T*_2_ with respect to temperature well above 100 °C. Therefore, it is probable that a chemical/or structural change is occurring in the castable’s porous network as the hydrates are transforming into more stable products in the higher temperature regime of 90–100 °C.

### Overall Temperature Correction

In an isothermal NMR experiment the water signal in a porous medium will decay multi-exponentially, reflecting the different pore states, i.e. *T*_2_ components of the system:

7 $$M(t) = \mathop \sum \limits_{i} m_{i} \exp \left( { - \frac{t}{{T_{2,i} }}} \right) .$$

To obtain the total quantitative moisture content for a temperature-induced drying experiment, the total NMR signal should incorporate effects from the magnetization as a function of temperature, and relaxation as a function of both the temperature and hydration. In conjunction with Eq. (1) we get the following:

8 $$M\left( {t,T} \right) = \mathop \sum \limits_{i} \left( {A_{c} (T)m_{i} } \right)A_{i} (T)\exp \left( { - \frac{t}{{T_{2,i} \left( T \right)}}} \right) ,$$

where *A*_*i*_ and *A*_c_ correspond to temperature correction for both the Arrhenius and Curie law, respectively, and *m*_*i*_ is the signal amplitude for the *i*th pore system. For castables, the *T*_2_ correction is dependent on both temperature and hydration, which requires measuring *T*_2_ as a function of temperature and hydration for every profile. Accordingly, the measured temperature profiles based on the six thermocouples are furthermore interpolated for a greater number of points that will supply the correction function.

**Fig. 5:**
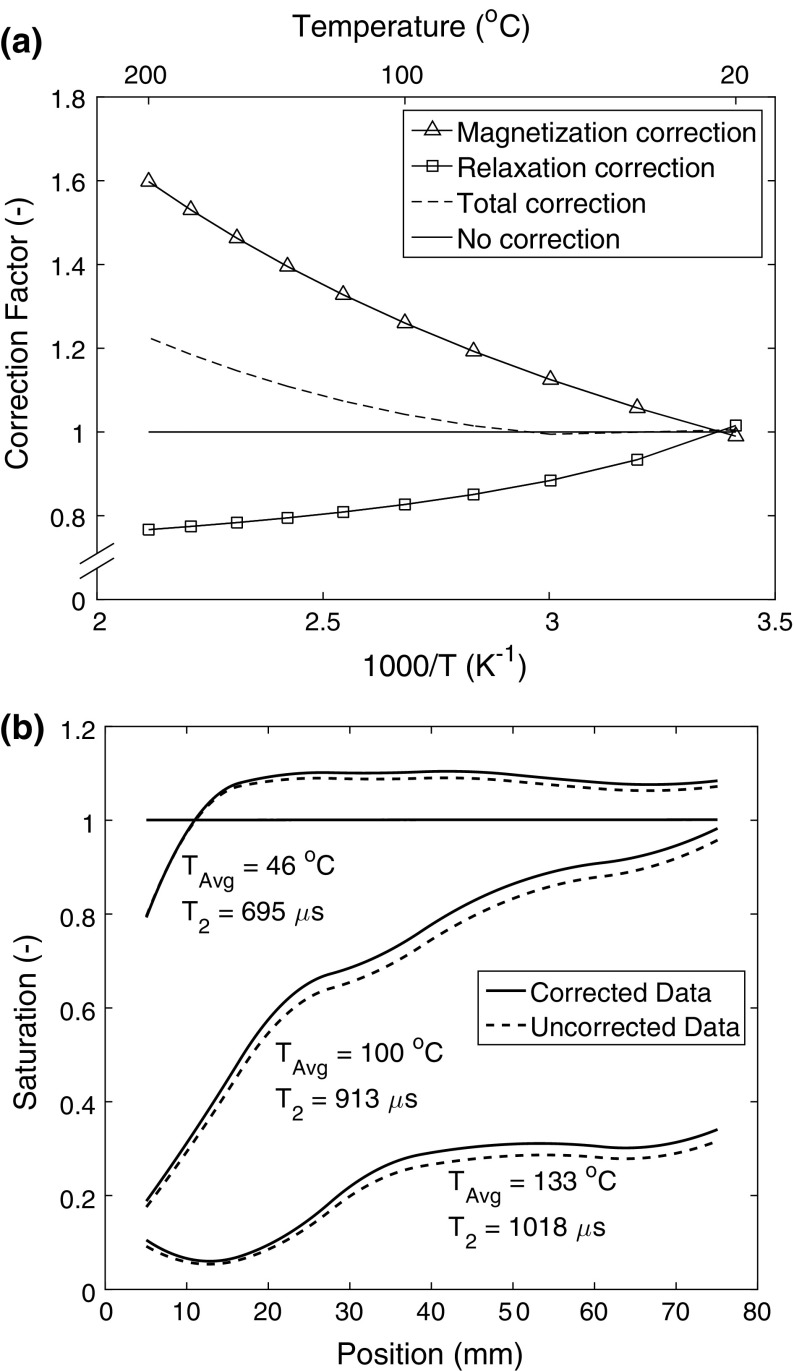
**a** Total temperature correction of the NMR signal for a sample cured for 48 h. The contributions from both the relaxation and magnetization effects are included. The solid line corresponds to no correction to the signal. **b** Selected moisture profiles (raw uncorrected + corrected) for an experiment with conventional castables cured for 48 h plotted next to each other. The corrected profiles are a sum of all different terms: magnetization (temperature) and relaxation (temperature, hydration state). The relaxation value is changing with every profile

As an example, in Fig. 5A we have shown the correction to the Curie law and *T*_2_, along with the total correction for the signal. Furthermore, the correction to the Curie law is independent of the material’s properties, whereas the *T*_2_ correction depends on the temperature and pore structure.

As can be seen for the total correction of both the magnetization and relaxation, the maximum correction (i.e. highest temperature) for the shortest $T_{2}$ components is just 20 per cent. The maximum total correction, including both relaxation and magnetization, at the highest temperatures will be 25–30%.

As an example, in Fig. 5b, we have plotted the uncorrected saturation for some selected profiles along with the total corrected profiles, i.e. for both temperature and hydration. Saturation is the raw data normalized to the first isothermal profile. The profiles illustrate that, as alluded to in the previous paragraph, the total correction to the degree of saturation is no more than 30%.

It is important to note that the correction only applies to the wet part of the sample, which is the fraction of pore space that is still saturated with liquid water. As the *T*_2_ calibration line corresponds to the relaxation dynamics of a saturated pore from *T* = 20–100 °C and since the signal drops to values below 10 per cent in the vapour region, the dry part of the sample does not significantly affect the relaxation correction. Indeed, it can be seen from Fig. 5b that the correction to the profiles in the front part of the sample is almost identical to the uncorrected profiles within the same region.

## First Drying of Castables

### Sample Preparation

The chosen sample for this study was a freshly prepared conventional castable cured for 48 h at room temperature. This particular curing time was chosen since it provides sufficient time for the calcium alumina binder to consume the water. The pre-mix is composed of tabular alumina (TA) grains which range in size from the micrometer to millimeter range. The pre-mix and binder constitute the total dry formulation (80/20% by mass), where the water demand is approximately 9% by mass (dry basis). The samples are dry mixed for 1 min and wet mixed for 4 min with a Hobart mixer, then subjected to mechanical vibration in order to achieve optimum densification of the porous matrix. The mixing time is fixed in order to ensure homogenisation and dispersion of water throughout the solid skeleton. After the samples have been vibrated, they are cast into 8 × 8 cm cylindrical Teflon moulds and covered overnight. The samples are 7.4 cm in length and 7.4 cm in diameter.

Furthermore, the solenoidal RF mini-coil is casted into the sample at the center, back-end. This means that the coil is initially fixed inside the mould, where fresh material is directly poured into it, i.e. during the wet phase when the material hardens into and around the coil. After 48 h have passed, the coil is rigidly secured for performing high-temperature NMR measurements.

### Moisture Content During First-Drying

**Fig. 6:**
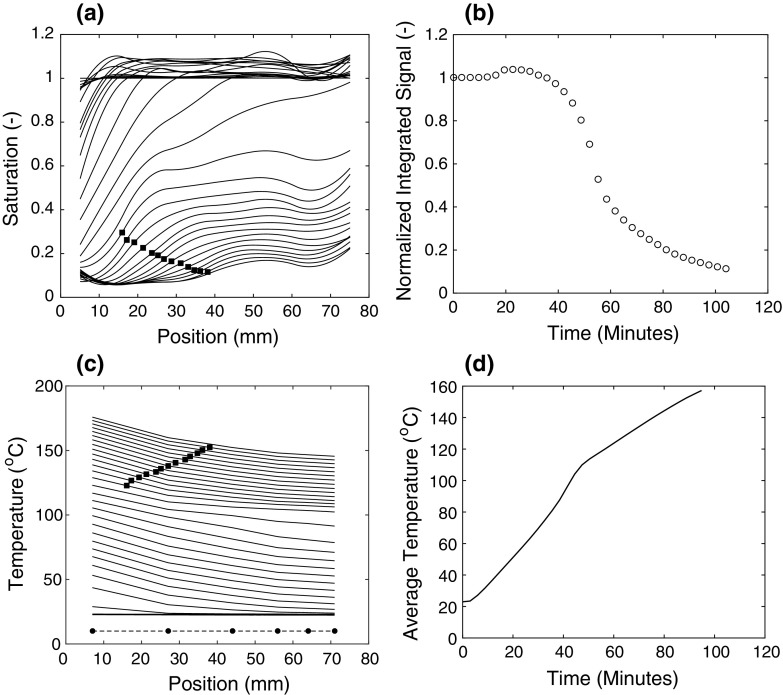
First-drying of a conventional castable cured for 48 h. The **a** NMR saturation profiles **b** total normalized saturation as a function of time **c** temperature profiles and **d** the average temperature as a function of time. The black square markers from **a** and **c** are a visual guide for the drying front in the saturation and temperature profiles. The six circular markers in **c** correspond to the thermocouple positions. The average time in between profiles is 3–4 min, with a total measuring time of 1.5 h

In Fig. 6, the results of the heating experiment are shown. Due to the sample length, the timescale of the experiment is around 1.5 h, after which there is no remaining signal. The temperature profiles were measured simultaneously along with the saturation profiles, with an average time of 3–4 min in between profiles. The temperature profiles reflect the conventional method of measuring heat transport during a first-drying experiment, where only superficial information is obtainable such as the temperature gradient and beginning of the boiling front. Figure 6c reveals that the boiling front begins around 100 °C, corresponding to the increase in energy intake of the sample.

Along with the temperature profiles, the saturation profiles are simultaneously measured and plotted in Fig. 6a. The saturation profiles are generated by taking every raw profile and dividing it by a reference taken at the beginning of the experiment, prior to switching on the heat. This allows for eliminating inhomogeneities that may arise from the field. Depending on the number of averages used to improve the signal-to-noise ratio, the time between each profile is 3–4 min. The maximum surface temperature reached is around 180–200 °C. Beyond this temperature, there is no remaining NMR signal.

Initially upon heating, we observe a slight increase in the total moisture content by about 5 per cent (i.e. a minor shoulder at 20 min). This reflects the fact that the shortest components of chemically combined water can be released from the hydrated state (the CPMG amplitude also increases in this range), even after correcting the signal for temperature effects. This transformation is not complete (i.e. towards the more stable crystallized gibbsite Al_2_O_3_∙3H_2_O) until 110 °C, as shown by the $T_{2}$ data (i.e. where it rapidly drops off around 120 °C), noted in the earlier discussion. However, the most pronounced observation in the drying behaviour is displayed by the inhomogeneous moisture distribution almost immediately after initial heat-up as shown in Fig. 6. In this next stage, there is only drying at the top part of the sample. The moisture is then driven out very rapidly until about 50 min have passed, corresponding to the second stage of drying. These first-two stages of moisture loss fall into the externally limited period of drying, where ambient conditions, such as temperature and humidity, determine the speed of drying.

However, after about 50% of the total moisture content has been removed from the sample, the drying front develops and slowly penetrates the pores (indicated by the markers in Fig. 6a, c). This slower stage of drying is known as the internally limited period and is governed by internal transport processes that reflect the pore structure and material characteristics of the sample, e.g. the ebullition and dehydration stages. The critical moisture content is defined as the boundary separating these characteristic stages of drying. It is only for quantities below the critical moisture content that the drying front emerges and travels through the sample.

It is also this lower range of moisture content that is most problematic from an industrial point of view, since the residual moisture must be slowly released as the corresponding steam pressures within the pores become aggressively high (depending on specific material characteristics). For conventional castables, the temperature of the front begins at 100 °C and does not exceed 150 °C. Therefore, the maximum pressure (based on the saturated vapor pressure curve) at the drying front is only 5 bars (0.5 MPa). These pressures are not significantly high compared to the strength of the material (≫ 1 MPa).

## Conclusions

In this study we have demonstrated and presented the functionality of a high-temperature NMR setup capable of performing non-destructive measurements on conventional castables during first-heat up. Due to the influence of changing temperature on transverse relaxation and magnetization, we have shown that the scale of the total correction factor to our signal is on the order of 25–30 per cent of the degree of saturation. Therefore, the $T_{2}$ relaxation has to be measured at the same time as the temperature for each point in the sample. Furthermore, we demonstrate the possibility of measuring quantitative moisture content profiles during drying of conventional castables by NMR, thereby providing direct observation of transport processes that are otherwise unobservable by destructive methods.

Applying the correction scheme to a first-drying experiment on a conventional castable cured for 48 h reveals that there are two principal drying stages: externally limited and internally limited drying; in the former case fast, environment-driven drying is observed, whereas in the latter case slow transport-driven drying is observed. Furthermore, the internally limited drying period correlates with the development of a drying front that penetrates the pores in the temperature range of 120–150 °C at the front position. By the time the maximum temperature of the front is reached, the average temperature across the sample is just under 200 °C.
